# Platelet-Therapeutics to Improve Tissue Regeneration and Wound Healing—Physiological Background and Methods of Preparation

**DOI:** 10.3390/biomedicines9080869

**Published:** 2021-07-22

**Authors:** Ellen E. Jansen, Andreas Braun, Patrick Jansen, Matthias Hartmann

**Affiliations:** 1 Clinic for Operative Dentistry, Periodontology and Preventive Dentistry, RWTH Aachen University, 52074 Aachen, Germany; ejansen@ukaachen.de (E.E.J.); anbraun@ukaachen.de (A.B.); pjansen@ukaachen.de (P.J.); 2 Klinik für Anästhesiologie und Intensivmedizin, Universitätsklinikum Essen, Universität Duisburg-Essen, 45122 Essen, Germany

**Keywords:** platelet-rich plasma, platelet-rich fibrin, tissue regeneration, wound healing

## Abstract

Besides their function in primary hemostasis, platelets are critically involved in the physiological steps leading to wound healing and tissue repair. For this purpose, platelets have a complex set of receptors allowing the recognition, binding, and manipulation of extracellular structures and the detection of pathogens and tissue damage. Intracellular vesicles contain a huge set of mediators that can be released to the extracellular space to coordinate the action of platelets as other cell types for tissue repair. Therapeutically, the most frequent use of platelets is the intravenous application of platelet concentrates in case of thrombocytopenia or thrombocytopathy. However, there is increasing evidence that the local application of platelet-rich concentrates and platelet-rich fibrin can improve wound healing and tissue repair in various settings in medicine and dentistry. For the therapeutic use of platelets in wound healing, several preparations are available in clinical practice. In the present study we discuss the physiology and the cellular mechanisms of platelets in hemostasis and wound repair, the methods used for the preparation of platelet-rich concentrates and platelet-rich fibrin, and highlight some examples of the therapeutic use in medicine and dentistry.

## 1. Introduction

In case of vascular injury, platelets detect the presence of subendothelial structures (extracellular matrix components) leading to the adhesion and aggregation of the anuclear cell type and primary hemostasis [1,2]. Secondary, platelets induce the activation of plasmatic coagulation and hemostasis. For the description of these complex temporal and spatial sequence of events occurring on the platelets’ surface, the cell-based coagulation model was developed [3]. Subsequent to clot formation, a further important mechanism of platelets takes place: activated GPIIb/IIIa receptors located on filopodia bind to fibrin, resulting in outside-in signaling of platelets and the activation of the contractile apparatus [4,5,6,7]. As a result, the platelets’ filopodia pull at the fibrin fibers to retract the clot [8]. Interestingly, the volume is reduced by the platelets action to at least 50% of volume and is inhibited when the red blood cells are tightly packed [9]. The huge effect of clot retraction has important physiological consequences: the size of the wound is retracted, the firmness of the clot is increased, densely packed polyhedral red blood cells form an impermeable membrane, and ischemia due to thrombosis of a vessel can be resolved by reperfusion [9,10,11]. In addition to their function in hemostasis, the clot consisting of platelets, leukocytes, fibrinogen, and erythrocytes coordinates inflammation and wound healing [12,13]. Inflammation is an important consequence of hemostasis, as injury can potentially be accompanied by the entry of pathogens. Besides leukocytes, platelets are an important and early step in the immune response to danger and infection and constitute an important cell type of both innate and adaptive immunity [2,14]. Platelets can detect pathogens and danger via pathogen recognition receptors, bind bacteria and viruses, induce the release of bactericidal NETs from neutrophils, and release platelet microbial proteins when stimulated with thrombin or lipopolysaccharides [14,15]. A further step initiated by platelets is wound repair, propagated by the release of various growth factors from alpha-granules attracting several different cell types necessary for wound healing. In the following, we will highlight the different functions of platelets, from vascular injury to wound healing, and will discuss the involvement in a platelet-centered approach. Moreover, we will discuss the preparation technique and the current use of platelet-rich plasma for local therapies in humans.

## 2. Platelet Physiology

Platelets are small anuclear cells with a short half-life of 10 days derived from megakaryocytes [16]. Platelets have a uniform discoid shape, a complex intracellular structure with a cytoskeleton maintained by dynamic action, can synthesize proteins, are capable of dividing, and can respond to various stimuli with shape change, adhesion, aggregation as well as exposure of phosphatidylserine on the surface to induce clot formation and hemostasis [17,18,19]. For a long time, the importance of these cells in hemostasis was acknowledged and platelet concentrates are nowadays a valuable treatment option in bleeding patients with thrombocytopenia or thrombocytopathy [20]. Besides this function, the involvement in wound healing and immune system function has been recognized. Thus, platelets have been demonstrated to affect inflammation, thrombosis, atherosclerosis, and metastasis [2,21,22].

For their diverse functions, platelets are equipped with multiple receptors to recognize their environment [23]. Physiological answers are achieved by inside-out signaling, leading to the important activation of the GPIIb/IIIa receptor (αIIbβ3) and to changes of the cytoskeleton, as well as the release of hundreds of mediators stored in intracellular granules. Moreover, platelets can communicate with other cells via extracellular vesicles (EV) and 90% of blood stream vesicles, which were named platelet dust in earlier times, derive from this cell type [24].

### 2.1. Platelet Granules and Mediators

Platelet mediators are released in alpha-granules, delta-granules (dense bodies), and lambda granules [8,25,26,27].

Alpha granules are described to release more than 300 proteins responsible for coagulation, anticoagulation, and fibrinolysis as well as being involved in inflammation, immunity, cell adhesion, and growth [14]. Among the mediators involved in coagulation are the factors V, XIII, and IX, fibrinogen, and the von Willebrand factor. Anticoagulant proteins include antithrombin, protein S, and tissue factor pathway inhibitors. Moreover, plasminogen and plasminogen activator inhibitor, proteins involved in fibrinolysis, can be released. Mediators involved in the recruitment of immune cells are the chemokines CXCL1, epithelial neutrophil activating peptide-78, platelet factor 4, monocyte chemoattractant protein-1, macrophage inflammatory protein 1 alpha, thymus- and activation-regulated chemokine (TARC), and regulated on activation, normal T cell expressed and secreted (RANTES = CCL5), as well as integral membrane proteins GPIIb/IIIa, GPIbalpha-IX-V, GPVI, TLT-1, and P-selectin. Moreover, many growth factors including platelet derived growth factor (PDGF), connective tissue growth factor (CTGF), stromal-derived factor-1 alpha, vascular endothelial growth factor (VEGF), tumor growth factors (TGFalpha, TGFbeta), and the fibroblast growth factor FGF-1, as well as the microbicidal proteins thymosin-beta4 and thrombocidin 1 and 2 can be released.

In contrast to alpha-granules, both the content and the function of delta-granules (dense bodies) are far less diverse, and the bioactive amines (serotonin, histamine), nucleotides, and poly- and pyrophosphates are all involved in clot formation and coagulation.

Lambda-granules are comparable to lysozymes in other cell type, are responsible for the degradation of proteins, lipids, and carbohydrates, and are thus involved in the removal of cell debris [28].

### 2.2. Extracellular Vesicles

An interesting new area of research are platelet derived extracellular vesicles. They are classified in micro-vesicles (100–1000 nm) and exosomes (30–100 nm), which are generated by different mechanisms (fusion of multi-vesicular bodies vs. budding from plasma membrane) and can be differentiated by cell surface markers [29]. They contain proteins, lipids, metabolites, miRNA, and nucleic acids and are involved in cell crosstalk and are thus involved in coagulation, inflammation, immunoregulation, and angiogenesis. Moreover, EVs have important functions in tissue repair and may exert the beneficial effects of platelet-rich plasma used in humans. In this regard, this beneficial effect can be “highjacked” by cancer cells for development and progression.

## 3. Platelets in Hemostasis

Hemostasis can be divided into three stages. Initially, vasoconstriction occurs at the side of vessel injury. Thereafter, a platelet plug is formed at the place of injury, a phenomenon called primary hemostasis. Secondary hemostasis leads to activation of coagulation system and the typical thrombus formation.

Under physiological conditions, resting platelets circulate in the blood stream to detect disturbances of vascular integrity. In case of vascular injury, platelets bind to von Willebrand factor via the glycoprotein GP Ibalpha of the GPIb-IX-V complex and GPVI. In turn, these events activate platelets GPIIb/IIIa, inducing aggregation and stable binding of platelets to the injury via fibrin and von Willebrand factor [1,4,30]. Moreover, platelet activation leads to the release of granules with pro-aggregatory and pro-coagulatory content, and tissue factor exposition on extravascular cells initiates further activation of platelets.

### 3.1. The Classical View on the Coagulation System

**Figure 1:**
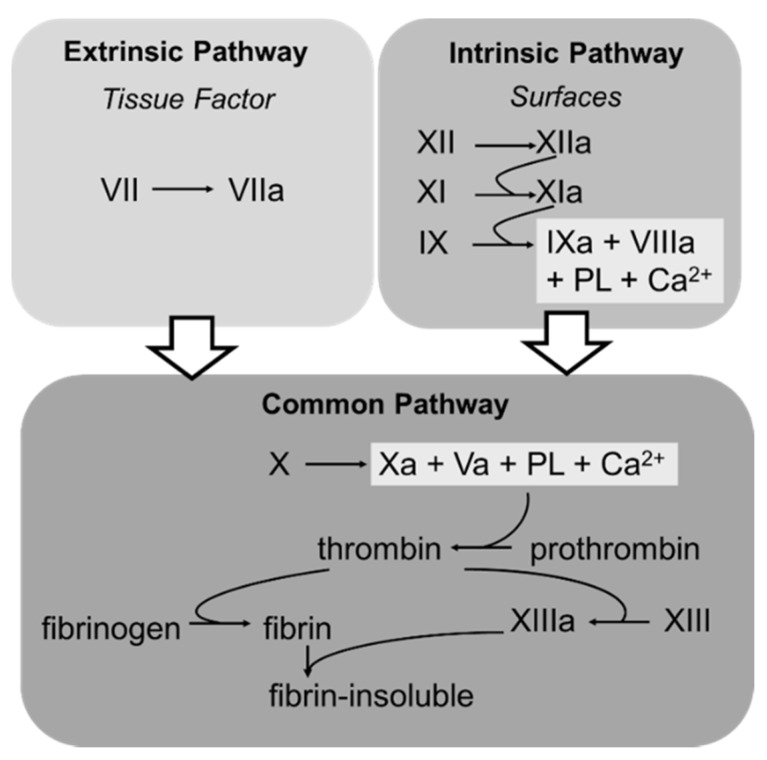
Schematic illustration of the intrinsic, extrinsic, and common pathway of the coagulation system.

In an initial attempt to understand the cooperative function of coagulation factors leading to hemostasis, the coagulation factors were initially described as an enzyme cascade leading to the generation of fibrin and hemostasis [31,32]. The coagulation factors were grouped in an intrinsic and an extrinsic pathway, which converged to the common pathway. The details are shown in Figure 1. While this classical model is very suitable to classify the effects of drugs and coagulation defects, it does not reflect the in vivo conditions and the important contribution of platelets.

### 3.2. The Cell-Based Coagulation Model

**Figure 2:**
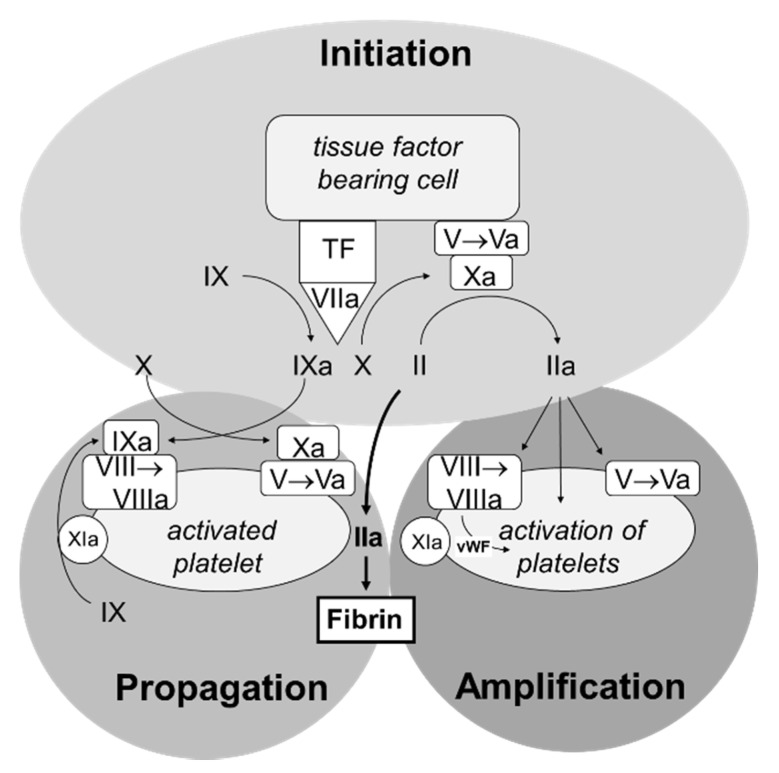
Cell-based coagulation model showing the contribution of tissue factor bearing cells and platelets in hemostasis.

To describe the concerted action of coagulation factors and platelets more exactly, the cell-based coagulation model has been proposed [3] which is shown in Figure 2. Under physiological circumstances, coagulation factors are restricted to the vascular space by the endothelium and have no contact to extravascular cells commonly expressing tissue factor on their surface. Vascular injury leads to the exposition of tissue factor bearing cells to the coagulation factors. Factor VII binds to tissue factor, is activated, and in turn activates the coagulation cascade. The minute amounts of thrombin are capable in activating platelets in the amplification phase. In the third step, the propagation phase, large amounts of thrombin are generated on the surface of the activated platelets sufficient to form a fibrin clot.

The cell-based coagulation system is a simplification which does not include the function of red blood cells in hemostasis [12]. Red blood cells improve hemostasis through an increase of blood viscosity, cause the margination of platelets in the blood stream, can adhere to the endothelium, and can thus favor thrombotic events under certain pathological conditions (e.g., diabetes). Moreover, red blood cells are involved in NO-metabolism, can release thromboxane A2 and ADP, and thus affect platelet aggregation and adhesion. Hemolysis of red blood cells and release of hemoglobin, generating ROS, can further activate coagulation and platelet activation. In addition to red blood cells, leukocytes have been shown to be involved in coagulation, thrombosis, and tissue damage [33].

### 3.3. Control of Hemostasis

There are several important pathways controlling coagulation. Thrombin, built during coagulation at the side of vascular injury, binds to endothelial thrombomodulin. The complex activates protein C which, in turn, inactivates the activated coagulation factors V an VIII:C. Protein S, which is the cofactor of protein C, assists the downregulation of coagulation [34]. Antithrombin is the most important circulating inhibitor of coagulation and binds and inactivates thrombin as well as several coagulation factors [35].

In case of clot formation, tissue plasminogen activator is released at the site of injury and converts plasminogen to plasmin [36]. Plasmin, in turn, degrades fibrin to fibrin degradation products. The activity of the fibrinolytic system is modulated by plasminogen activator inhibitor I and II, plasmin inhibitor, and thrombin-activatable fibrinolysis inhibitor.

## 4. Platelets in Clot Retraction

Subsequent to the formation of fibrin fibers, GPIIb/IIIA receptors located on the filopodiae of the activated platelets bind to fibrin fibers and induce via outside-in signaling the activation of the contractile apparatus [9,10,11]. As a result, platelets pull at the fibrin fibers and lead to the contraction of the clot. Clot retraction is limited when red blood cells are compacted [37]. While the cellular mechanisms of signal-transduction and the contractile mechanisms have been investigated in detail, only sparse information in humans is available. It is generally accepted that clot retraction is an important mechanism to (i) improve wound healing and to (ii) enable the reperfusion of a vessel in case of thrombosis [38]. Moreover, thrombasthenia Glanzmann-Naegeli and Bernhard-Soulier syndrome are characterized by a bleeding phenotype associated with disturbed clot retraction and defective GPIIb/IIIa receptors [8]. Only few information of altered clot retraction in acquired diseases is available: increased clot retraction has been demonstrated in coronary heart disease, while a decrease was shown in uremic patients [39,40].

## 5. Platelets in Immunology and Wound Healing

Wound healing starts with hemostasis, the formation of a fibrin scaffold, and an inflammatory response as a first line of defense with a recruitment of neutrophils and monocytes. Platelets are involved in both the innate immune system and adaptive immunity responses [41]. For this purpose, platelets are equipped with several pattern recognition receptors such as Toll-like receptors and C-type lectin receptors, which can detect pathogen associated molecular patterns and danger associated molecular patterns [42]. TLR4-induced activation of platelets results in the release of proinflammatory mediators, the recruitment of leukocytes, and aggregates of platelets with leukocytes or monocytes [15]. Leukocytes, in turn, release cytokines and chemokines to modulate inflammation. Moreover, neutrophils can release reactive oxygen species as well as their nuclear content to form neutrophil extracellular traps (NETs) to fight pathogens [43].

Besides their function in innate immunity, platelet CD40-ligand, expressed upon activation, can bind to many immune cell types, including B-cells, T-cells, and endothelial cells via their CD40-receptors [44]. In this way, platelets can modulate the release of cytokine and immunoglobulin production.

Subsequent to this initial phase necessary to eliminate pathogens, angiogenesis occurs, which includes endothelial cell proliferation, migration, and branching of vessels [45]. Moreover, pericytes as well as all other cell types of the perivascular space proliferate. In addition, circulating progenitor cells from the bone marrow support new vessel formation. During the development of blood vessels, fibroblasts proliferate and invade into the clot and shift the cellular environment from the inflammatory to a growth state. Differentiation of some fibroblasts to myofibroblasts leads to a further retraction of the wound and finally to scar formation, in parallel to re-epithelialization. Platelets have been involved in the whole process of healing via the release of multiple growth factors in their secretome [46]. Increased platelet concentration using platelet-rich plasma in the wound has regularly improved wound healing in several animal models and is FDA approved. However, thrombocytopenia in a mouse model did not affect wound healing as judged by angiogenesis, collagen synthesis, and re-epithelialization [47].

## 6. Preparation of Platelet-Rich Plasma and Platelet-Rich Fibrin

**Figure 3:**
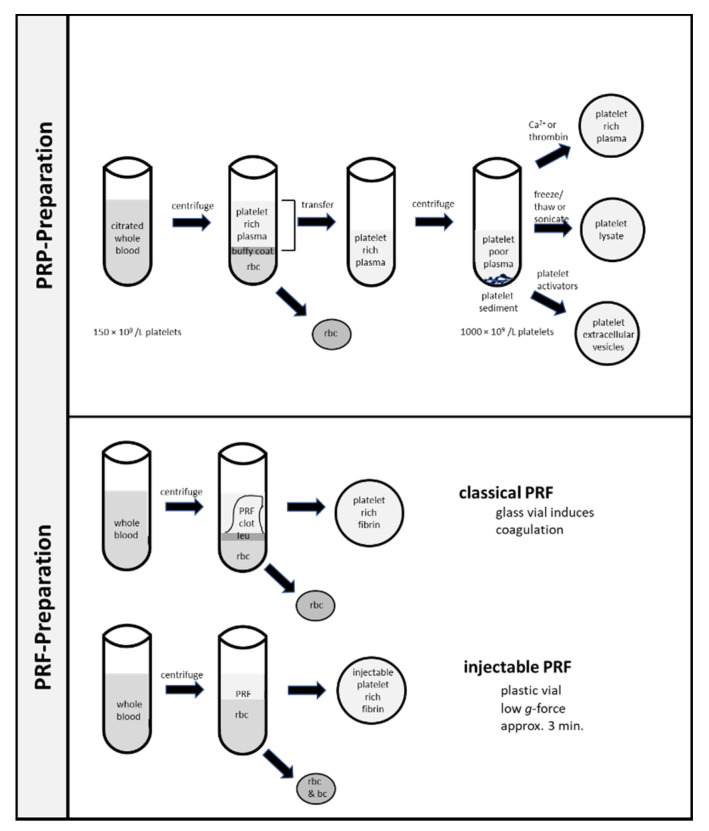
Methods for the preparation of platelet-rich concentrates. Initial steps for the preparation of platelet-rich plasma, platelet-rich lysate, and platelet vesicles are identical. Note, that both classical platelet-rich fibrin and injectable platelet-rich fibrin can be obtained by a single centrifugation without the necessity of anticoagulation; leu: leukocytes; rbc: red blood cells; bc: buffy coat.

The rational for the use of blood based local therapy relies on the finding that a blood clot improves wound healing by the release of growth factors, chemokines, and antibiotic agents from platelets [14,48]. An increase of platelet count in the wound was shown to enhance wound healing in an experimental setting. While platelets are shown to be an important source of mediators, there is ample evidence that the other constituents of blood, including leukocytes and fibrin, can also contribute to wound healing. Leukocytes are important for local defense and are capable in releasing growth factors (similar to platelets). However, the eventual harms due to their inflammatory action is an object of the debate. Moreover, the fibrin mesh serves as a scaffold for immune cells, fibrocytes, and stem cells. Different preparation methods of platelet concentrates are shown in Figure 3.

### 6.1. Platelet Rich Plasma

In 1997, autologous platelet-rich plasma was first used in oral and maxillofacial surgery [49]. For the preparation, anticoagulated whole blood is centrifuged to separate red blood cells from platelets [27]. Red blood cells sediment at the bottom of the vial and plasma is located at the top. Between red blood cells and plasma there is a small layer called the buffy coat containing leukocytes and platelets. Depending on the conditions of centrifugation, the plasma contains a variable number of platelets. The plasma containing platelets is collected, sedimented in a second centrifugation step, and reconstituted in a defined amount of plasma. Thereafter, coagulation is initiated by the addition of Ca^++^ or thrombin [50,51]. The aim of the centrifugation steps is the enrichment of platelets (as well as leukocytes) to enhance the therapeutic effect. Typically, an enrichment of platelets from 150 × 10^9^–350 × 10^9^/L in whole blood to about 1000 × 10^9^/L in platelet-rich plasma are judged to be advantageous [50]. Meanwhile, more than 40 different procedures for the preparation of platelet-rich plasma are described [50].

### 6.2. Platelet Rich Fibrin

In 2006, Choukroun et al. described the use of another platelet preparation obtained in a single centrifugation step and called it platelet-rich fibrin [52,53,54,55,56]. The procedure includes a single centrifugation (400× *g*; 12 min) of whole blood without anticoagulation in a glass vial. After a single centrifugation step, the red blood sediment is separated from a clot formed during centrifugation containing enriched platelets, as well as leukocytes at the border line. The simpler handling with only one centrifugation step and without the need of thrombin addition is the important advantage of this method. Indeed, this method is widely used in clinical medicine. Meanwhile, several different centrifugation protocols are available, which show different enrichment in platelet and leukocyte count as recently reviewed [48]. For the preparation enriched in platelets, centrifugation of whole blood samples is used. In order to increase or delay coagulation and thus the formation of the fibrin mesh, centrifugation is performed in either glass or plastic tubes. Centrifugation with 60–1200× *g* for 3 to 15 min leads to a separation of cell types due to the different physical properties [57]. Red blood cells are sedimented at the bottom of the vial. Located above the red blood cell sediment, there is a small layer named the buffy coat which consists of platelets and leukocytes. On top of the buffy coat, the plasma is located. Depending on the centrifugation conditions, platelet concentration of plasma varies. The harsher the centrifugation conditions, the more platelets will sediment in the buffy coat. According to the different protocols, leukocyte-poor platelet-rich fibrin, leukocyte-rich fibrin, and liquid platelet rich fibrin can be differentiated [48,58].

### 6.3. Platelet Lysates

A third method claimed platelet lysate has recently been developed [59]. For this platelet product, platelet-rich plasma is treated by either freeze thaw cycles or sonication. The resulting platelet lysates can be stored frozen for an extended time. While the use in clinical medicine is relatively new, the preparation is often used as a source of growth factors in cell cultures.

### 6.4. Platelet Extracellular Vesicles

In the future, a fourth method for the use of platelet in clinical medicine might derive on the finding that activation of platelets leads to the mass release of extracellular vesicles, which could serve as the source of growth factors in local therapies [60]. Two types of extracellular vesicles can be differentiated: exosomes (30–100 nm) and microvesicles (100–1000 nm) [61]. There is no information available on the advantages and disadvantages of each preparation regimen in human use. However, different compositions have been demonstrated and may lead to different characteristics.

## 7. Therapeutic Use of Platelet Rich Concentrates in Human Diseases

Countless in vivo and in vitro studies exist on the clinical use of platelet concentrates in medicine, and there are more than three hundred reviews and meta-analyses on the clinical use in different settings. It is therefore beyond the scope and the possibilities of a single review to give in-depth information on the use of all subspecialities. According to most reviews and meta-analyses, the level of evidence is somewhat limited by the fact that small studies with few patients and a high risk of bias dominate the literature. Moreover, the use of many different platelet rich concentrates complicates the interpretation and comparison of the results, and the need for studies with standardized platelet-rich concentrates has been emphasized [62]. In this regard, the evidence for the use of other commonly used blood products, including red blood cell concentrates, platelet concentrates, fresh frozen plasma, and factor concentrates, is often limited in different settings and reflects that research is most often investigator-initiated studies with limited financial support. Thus, further well-designed prospective studies may be valuable to better judge the advantages and disadvantages of platelet concentrate based therapies and to find the best platelet preparation. However, despite these limitations, there seems to be sufficient evidence in the literature for the use of platelet-rich plasma and platelet-rich fibrin in many clinical settings.

### Platelet-Rich Concentrates in Medicine and Dentistry

Platelet concentrates are applied in many medical subdisciplines including sports medicine, plastic surgery, dermatology, otolaryngology, gynecology, urology, and diabetology, among others. Moreover, platelet-rich concentrates are widely used in dentistry and oral and maxillofacial surgery [63,64,65].

The use of platelet-rich concentrates in wound healing is well established [66,67]. Platelet-rich fibrin and platelet-rich plasma are used in chronic diabetic wounds as an efficient, economical, and simple adjuvant method to support tissue regeneration [68,69]. It can also be injected to treat scars such as acne or striae distensae [70,71]. Moreover, platelet-rich concentrates are increasingly used in androgenetic alopecia [72,73] as well as skin rejuvenation and skin augmentation [66,74].

Another area is the use in chronic, mostly degenerative pain conditions. In knee arthrosis, degenerative disc disease (intradiscal treatment), facet pathologies (intrafacial injection), and sacroiliitis, reduced pain scores and increased functionality were observed after platelet-rich plasma therapy [75]. Improvement can also be observed in severe temporomandibular joint disorders through platelet-rich fibrin injection after arthrocentesis into the upper join space or with adjuvant platelet-rich plasma insertion during arthroscopy procedures or arthrocentesis [76,77].

In dentistry, the application of platelet preparation is widely used for bone repair, a significantly faster healing of bony defects was determined radiologically after adjunctive use of platelet-rich fibrin [78].

Wide ranges of possible applications are also known in the treatment of periodontitis [65]. Bone defects can be filled with previously platelet-rich fibrin-inoculated substitutes, which leads to a significant reduction in probing depths [63,79,80]. In addition, the application of a platelet-rich fibrin membrane can prevent the ingrowth of epithelial cells into the treated bony defect and can promote the ingrowth of osteogenic and angiogenic cells [81,82]. The use of platelet-rich fibrin in the surgical treatment of Class II furcation appears to improve periodontal regeneration. In combination with bone graft substitutes, vertical clinical attachment loss was significantly reduced [83]. Moreover, improved regeneration of the periodontal attachment has been demonstrated [63,64,67]. Platelet-rich fibrin membranes are also considered a promising alternative for covering recessions; compared to subepithelial connective tissue grafts—the gold standard—no significant difference in gingival recessions, clinical attachment level, and probing depths were observed [63,84]. Thus, the results of the study indicate that the use of invasive procedures and the necessity of a graft can be avoided by platelet-rich fibrin.

Another application of platelet concentrates is the use in vitality-preserving endodontics. A positive influence of platelet-rich fibrin and platelet-rich plasma preparations on healing after vital amputation was observed. However, platelet-rich plasma seems to lead to less coronal discoloration. The combined use of platelet-rich plasma and mineral trioxide aggregate (MTA) showed a better prognosis compared to the use of MTA alone for apexogenesis [85,86]. Similarly, positive effects were observed with the use of platelet-rich plasma or platelet-rich fibrin compared to the most used therapeutic method of blood clot revascularization, for the regeneration of immature permanent teeth [87].

## 8. Conclusions

Platelet-rich plasma and platelet-rich fibrin are used as a source of various mediators, which favor hemostasis, wound healing, and tissue repair. The effects can be explained by the application of supernormal concentrations of various platelet-derived mediators, including many growth factors, chemokines, hemostatic and antibiotic peptides, as well as the fibrin mesh serving as a scaffold for repair. While there is much evidence that the platelet-concentrates are effective in many clinical settings, the advantages and disadvantages of different preparations are not known. Furthermore, there is a lack of standardization in respect to platelet count, leukocyte count, and fibrinogen.

## Data Availability

Not applicable.
